# Geographically structured genotypes and resistance clustering in *Aspergillus fumigatus*

**DOI:** 10.1007/s10096-025-05390-4

**Published:** 2025-12-24

**Authors:** Won-Bok Kim, Dukhee Nho, Sung-Yeon Cho, Dong-Gun Lee, Chulmin Park, Raeseok Lee

**Affiliations:** 1 Genes Laboratories Inc, Yongin-si, Gyeonggi-do Republic of Korea; 2 Division of Infectious Diseases, Department of Internal Medicine, College of Medicine, The Catholic University of Korea, Seoul, Republic of Korea; 3 Vaccine Bio Research Institute, College of Medicine, The Catholic University of Korea, Seoul, Republic of Korea

**Keywords:** Aspergillus fumigatus, Molecular epidemiology, Geographic distribution, Antifungal resistance, Environmental microbiology

## Abstract

We analysed 498 global *Aspergillus fumigatus* isolates using multilocus variable-number tandem-repeat (MLVA) typing to investigate regional clustering, environmental–clinical overlap, and azole resistance patterns. The dataset, which included 155 newly genotyped Korean strains, revealed extensive genotypic diversity and four distinct phylogeographic clusters. Resistance-associated mutations (TR34/TR46) appeared concentrated within certain clusters that largely comprised isolates from Germany, South Korea, and China, suggesting country-level enrichment rather than broader continental trends. These findings support the presence of geographically structured populations and localised emergence of resistance and demonstrate the utility of MLVA for molecular surveillance, particularly in settings where whole-genome sequencing is limited.

**Supplementary Information:**

The online version contains supplementary material available at 10.1007/s10096-025-05390-4.

## Introduction

Despite growing interest, key gaps exist in our understanding of *Aspergillus fumigatus* molecular epidemiology. Global-scale genotyping efforts are limited, and the population structure of this species is poorly defined across regions [1]. Although recent whole-genome sequencing (WGS)-based studies have shown that clinical isolates span a broad genetic spectrum rather than a single virulent lineage, the extent to which this diversity contributes to disease across different regions has not yet been unresolved [2, 3]. Additionally, the geographic distribution of these genotypes has not been systematically characterised; therefore, whether strains are globally dispersed or exhibit regional restriction is uncertain [4]. Finally, the emergence of azole-resistant isolates has raised urgent questions about how local environmental selection pressures may shape the distribution of resistance-associated genotypes [5].

Although WGS offers the highest resolution, its global application is often constrained by resource availability and metadata limitations, particularly across environmental sources. In such contexts, multilocus variable-number tandem-repeat (MLVA) typing is a practical and informative tool for large-scale genotypic surveillance [4, 6]. Previous MLVA-based global analyses, including the study by Xu et al., have provided early insights regarding the continental population structure and resistance-associated lineages [4].

In this study, we built upon these previous efforts by integrating all publicly available genome-derived MLVA profiles with recent clinical and environmental isolates from South Korea [7, 8]. We assessed regional genotypic clustering, overlap between environmental and clinical strains, and distributions of resistance-associated mutations to inform geographically tailored surveillance strategies and highlight the need for an integrated health framework that bridges clinical and environmental monitoring.

## Materials and methods

### Data collection and processing

We analysed 498 *A. fumigatus* isolates comprising 343 publicly available genomes (National Center for Biotechnology Information [NCBI] as of 30 April 2025) and 155 South Korean clinical/environmental isolates from previous studies [7, 8]. For the Korea isolates, conventional wet-lab MLVA profiles were adopted [7, 8]. For public genomes, MLVA alleles and *cyp51A* mutations were inferred in silico using a BLASTn-based workflow (NCBI BLAST; accessed April 2025). Genomes missing at least one target locus or metadata were excluded.

### Molecular typing and phylogenetic analysis

For each of the 10 MLVA loci, alleles were defined based on the sequence variation and converted into numeric codes. Sequence types (STs) were assigned by concatenating the 10-locus allele codes for each isolate. Genetic relatedness among STs was assessed using Euclidean distance and goeBURST-based algorithms. Minimal spanning trees (MSTs) were constructed using PHYLOViZ (version 2.0; Lisbon, Portugal) to visualise phylogenetic relationships. Because paired isolates with both conventional MLVA and WGS data were unavailable, direct in vitro–in silico concordance testing was not performed. Detailed bioinformatic protocols, including allele calling criteria and handling of ambiguous loci, are provided in the Supplementary methods.

### Discriminatory power and resistance analysis

The discriminatory capacity of the 10-locus MLVA scheme was assessed using Simpson’s diversity index [9]. Tandem-repeat (TR) mutations in the *cyp51A* promoter were assessed for all 498 isolates. For the 155 South Korean isolates, resistance data were adopted from previous in vitro studies [7, 8]. For the 343 public genomes, *cyp51A* promoter and coding sequences were re-identified using a standardized bioinformatic workflow (NCBI BLAST; accessed April 2025) rather than relying on existing annotations. The resistance status was defined based on the presence of TR34/TR46 mutations. Detailed calculation formulas and bioinformatic protocols are provided in the Supplementary methods.

## Results

### Genotypic diversity

A total of 498 *A. fumigatus* isolates comprising 343 publicly available genomes and 155 strains collected in South Korea were analysed. These isolates originated from 17 countries; however, the majority originated from Germany (256; 51.4%), South Korea (155; 31.1%), and China (43; 8.6%). Additional isolates originated from France (10), the United States (7), and the United Kingdom (7). Isolates from other countries with five or fewer samples each were also included. The detailed country-level distribution is provided in Supplementary Table S1, with isolate-level data available in the Supplementary File.

MLVA typing divided the 498 isolates into 343 distinct STs, thus demonstrating high discriminatory capacity; additionally, 271 STs (79%) were observed only once, indicating broad genotypic diversity. ST138 comprised 26 clinical isolates collected from China in 2019, suggesting a locally dominant lineage. Overall, the MLVA scheme exhibited very high discriminatory power with a Simpson’s diversity index of 0.995, thus reflecting the large number of unique sequence types and limited clustering of isolates within the same MLVA type (Supplementary File).

### Phylogenetic clustering and regional structure

**Fig. 1:**
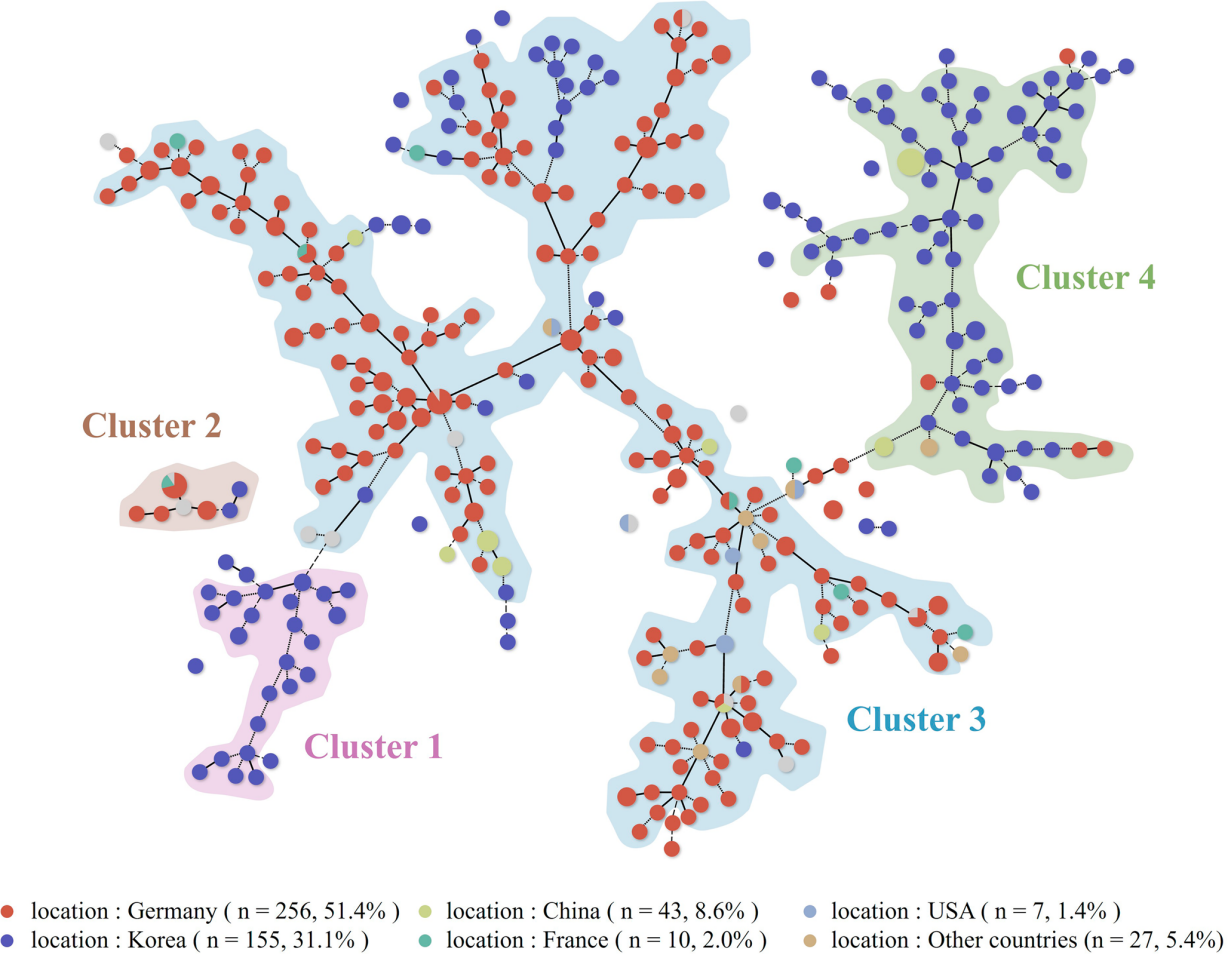
Minimum spanning tree of 498 *Aspergillus fumigatus* isolates based on multilocus variable-number tandem-repeat profiles. Each node represents a unique sequence type (ST). The node size is proportional to the number of isolates assigned to that ST. Nodes are colour-coded by country as follows: Germany, red; South Korea, blue; China, light green; France, lavender; USA, blue-grey; and other countries, light grey. Edges indicate genetic distances as follows: solid lines denote single-locus variants; short dashed lines indicate two-locus variants; and long dashed lines correspond to three-locus variants. Four major phylogenetic clusters are shaded to highlight regional genotypic structuring

The MST analysis revealed four major phylogenetic clusters. Clusters 1 and 4 largely comprised South Korean and Chinese isolates, whereas cluster 3 was dominated by strains from Germany and other European countries. This clustering pattern highlighted the regional stratification of genotypes, suggesting local evolutionary adaptation or restricted strain flow (Fig. 1).

### Environmental and clinical source distributions

Environmental and clinical isolates were broadly intermixed across all clusters without clear separation, thus indicating shared genotypes between environmental reservoirs and human infections (Figure S1).

### Azole resistance and genotypic aggregation

Twenty-one isolates (4.2%) harboured *cyp51A* promoter TR mutations (15 TR34 and 6 TR46 mutations) spanning 19 distinct STs. Resistance was geographically structured. TR34 mutations were predominantly found in Europe (15; 11 of these 15 were from Germany and Spain), whereas TR46 mutations were found in Europe (4) and South Korea (2). Notably, no TR-positive isolates were identified among the Chinese strains (Table S2). In the MST, resistance-associated STs were largely localized within Europe-dominated cluster 3, and a smaller subset was localized in Korea-dominated cluster 4 (Figure S2).

## Discussion

This global-scale genotypic analysis of *A. fumigatus* using MLVA profiling revealed distinct regional population structures. Despite the airborne nature of *A. fumigatus* and its presumed global dispersion, our findings showed clear phylogeographic clustering. South Korean and Chinese isolates formed distinct lineages from the European clusters, which were dominated by strains from Germany and France. These results support the presence of geographically enriched genotypes and suggest local clonal expansion or microevolutionary adaptation, consistent with the results of recent pan-genomic studies that found restricted gene flow and region-specific genomic divergence in *A. fumigatus* [10]. Such region-specific lineage structuring suggests local clonal expansion or niche-driven microevolution, thus reinforcing the need for tailored molecular surveillance appropriate for each geographic region. These findings are broadly consistent with those of the global MLVA analysis by Xu et al. that also identified major continental lineages. Our study extended this work by incorporating more recent East Asian and European isolates and applying updated MLVA profiles, thus providing a contemporary view of the genotype distribution and resistance-associated clustering [4].

A genetic analysis revealed extensive intermixing of clinical and environmental isolates across all phylogenetic clusters without source-specific lineage segregation. The extensive overlap between environmental and clinical isolates across all phylogenetic clusters suggested shared reservoirs and circulation of potentially pathogenic genotypes in the environment [7, 11]. These findings reinforce the importance of environmental surveillance as part of a One Health framework that links clinical disease dynamics to environmental reservoirs.

Azole resistance-associated STs were not evenly distributed, but they exhibited a regional pattern. Of the 19 STs that carried *cyp51A* TR mutations (TR34 or TR46), 13 (68.4%) were located within cluster 3, which primarily comprised isolates from Germany and other European countries. This trend aligns with the findings of our quantitative analysis, which showed that 73% of TR34-positive isolates originated from Europe and TR46 mutations were more evenly distributed between Europe and East Asia. This regional pattern is also consistent with the findings of prior studies that reported more frequent TR34 mutations in European isolates [12]. The remaining STs, including TR46-positive ST109 and ST123, were found in cluster 1, which was dominated by isolates from South Korea. Although sampling imbalances and underreporting in other regions cannot be excluded, this distribution indicates that resistance-associated genotypes are concentrated within certain geographic lineages. Although the underlying drivers of this pattern require confirmation, these observations support the importance of regionally focused antifungal resistance surveillance and molecular monitoring programmes [13–15].

This study had some limitations. First, sampling was geographically uneven. More than 80% of isolates were from Germany and South Korea, which have influenced cluster boundaries. Second, combining conventional (Korea) and WGS-derived (global) MLVA profiles prevented direct isolate-by-isolate concordance validation. Although manual verification was applied to representative genotypes to mitigate artifacts from short-read assemblies, fine-scale phylogeographic interpretations require caution [16]. Finally, our analysis focused on promoter TR mutations. Other clinically relevant *cyp51A* mutations (e.g., L98H, Y121F) and non-*cyp51A* mechanisms were not assessed; therefore, future longitudinal studies that incorporate broader coding region mutations are necessary to fully characterise resistance dynamics.

## Conclusion

*A. fumigatus* demonstrates a regional population structure, environmental–clinical genetic overlap, and localised clustering of resistance-associated genotypes. These findings highlight the need for geographically informed molecular surveillance, integrated antifungal stewardship, and longitudinal epidemiological studies. Sustained genomic data sharing across clinical and environmental domains are essential to supporting a health-based strategy that enables early detection and containment of emerging resistance.

## Supplementary Information

Below is the link to the electronic supplementary material.

Supplementary Material 1

Supplementary Material 2

## Data Availability

The datasets generated and analysed in this study, including the detailed MLVA profiles and resistance data, are provided in the Supplementary Files submitted for this article and are available for research use.
