# Supplementary material for: Geographically structured genotypes and resistance clustering in Aspergillus fumigatus
Source: Eur J Clin Microbiol Infect Dis. 2025 Dec 24;45(4):1201–5. doi: 10.1007/s10096-025-05390-4 (PMC13086806; doi:10.1007/s10096-025-05390-4)
Supplement: Supplementary file 1 — Supplementary Material 1 [file 10096_2025_5390_MOESM1_ESM.docx]

**Supplementary material**

**Brief Reports**

**Geographically structured genotypes and resistance clustering in *Aspergillus fumigatus***

Won-Bok Kim, Dukhee Nho, Sung-Yeon Cho, Dong-Gun Lee, Chulmin Park, Raeseok Lee

**^*^Corresponding author: Raeseok Lee, MD, MPH, PhD**

Division of Infectious Diseases, Department of Internal Medicine, Seoul St. Mary’s Hospital, College of Medicine, The Catholic University of Korea, 222, Banpo-daero, Seocho-gu, Seoul, 06591, Republic of Korea

Tel: +82-2-2258-6751, Fax: +82-2-785-7944, E-mail: misozium03@catholic.ac.kr

ORCID: 0000-0002-1168-3666

**Table of contents:**

**Supplementary method ……………………………………………………………………**3

**Table S1.** Geographic distribution and number of distinct sequence types of *Aspergillus fumigatus* isolates**……………………………………………………………………………**6

**Table S2.** Geographic and genotypic distribution of *cyp51A* promotor tandem-repeat mutations among azole-resistant *Aspergillus fumigatus* isolates **…………………………...**7

**Figure S1.** Minimum spanning tree of 498 *Aspergillus fumigatus* isolates, color-coded by source of isolation **……………………………………………………………………………**8

**Figure S2.** Minimum spanning tree of 498 *Aspergillus fumigatus* isolates highlighting *cyp51A* promotor tandem-repeat mutations associated with azole resistance**. ………………**9

**References …………………………………………………………………………………**10

**Supplementary method**

***Genome Selection Criteria***

To construct a comprehensive global dataset, we queried the NCBI database (as of 30 April 2025) for publicly available *Aspergillus fumigatus* whole-genome sequences. Candidate genomes were screened in silico using BLASTn against the ten multilocus variable-number tandem-repeat (MLVA) loci to ensure typing feasibility. Only genomes in which all ten loci were detectable and for which essential metadata (source and country of origin) were available were retained. Genomes missing ≥1 target locus or lacking sufficient metadata were excluded from the study. Consequently, a total of 343 publicly available genomes were selected and combined with 155 South Korean clinical and environmental isolates from our previous studies, resulting in a final dataset of 498 isolates [1, 2].

***In silico MLVA Typing***

For the 343 publicly available genomes, MLVA alleles were inferred in silico. This approach aligns with previous studies that derived MLVA genotypes from whole-genome sequencing (WGS) data while maintaining backward compatibility with traditional typing schemes [3, 4]. We utilised the NCBI BLASTn web interface (accessed April 2025, default parameters) with locus-specific reference sequences for each of the 10 VNTR loci. The best hit was used to retrieve the corresponding tandem-repeat (TR) region from each assembly, and the number of repeat units was determined by counting occurrences of the locus-specific repeat motif within the extracted region. Since direct in vitro–in silico concordance testing was not possible due to the lack of paired isolates, we adopted this established in silico workflow with manual verification. For representative genotypes and TR-positive strains, alignments were manually inspected, and repeat counts were confirmed by visual counting. If BLASTn did not return a clear hit or the alignment was ambiguous, the locus was coded as “0” (missing) and the isolate was excluded from downstream diversity analyses.

***Assessment of Azole Resistance (cyp51A Analysis)***

For the 343 publicly available genomes, we re-identified *cyp51A* mutations using a standardized workflow to ensure consistency, rather than relying on existing database annotations. The *cyp51A* locus was extracted from each assembly using the NCBI BLASTn web interface (accessed April 2025, default parameters) with locus-specific reference sequences. Extracted sequences were aligned to a wild-type *cyp51A* reference. We screened the promoter region for TR34 and TR46 insertions and the coding region for known azole resistance–associated substitutions (e.g., L98H, Y121F). In our dataset, isolates carrying key coding-region substitutions consistently also harboured promoter TR mutations; therefore, resistance status for the MLVA analysis was defined based on the presence of TR34 or TR46 (classified as TR34, TR46, or no promoter TR detected).

***Discriminatory Power Calculation***

The discriminatory capacity of the MLVA scheme was calculated from the distribution of sequence types across all isolates using the Hunter–Gaston modification of Simpson’s diversity index, according to the formula:


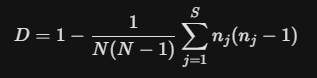


N is the total number of isolates, S is the total number of described sequence types, and nj is the number of isolates belonging to the j-th sequence type [5]. The index was calculated based on the combined 10-locus profiles rather than averaging locus-specific indices.

**Table S1.** Geographic distribution and number of distinct sequence types (STs) of *Aspergillus fumigatus* isolates

| Location | Isolates  count | Number of  distinct STs |
| --- | --- | --- |
| Australia | 2 | 2 |
| Austria | 2 | 2 |
| Brazil | 1 | 1 |
| Canada | 2 | 2 |
| China | 43 | 9 |
| France | 10 | 8 |
| Germany | 256 | 176 |
| Japan | 1 | 1^a^ |
| Korea | 155 | 131 |
| Netherlands | 1 | 1 |
| New Zealand | 2 | 2 |
| Peru | 1 | 1 |
| Russia | 1 | 1 |
| Space | 2 | 2^b^ |
| Spain | 5 | 5 |
| United Kingdom | 7 | 6 |
| USA | 7 | 6 |
| Total | 498 | 356(343)^c^ |

^a^ JCM 10253 is registered in Japan (NCBI), but was originally isolated from a human lung in the USA (listed as “animal” in the database).

^b^ Strains IF1SW-F4 and ISSFT-021 were isolated from the International Space Station; isolation and analysis were conducted by NASA.

^c^ Aggregating ST types by country yields 356 STs; however, 13 STs are shared between countries, resulting in a total of 343 distinct STs.

**Table S2.** Geographic and genotypic distribution of *cyp51A* promotor tandem-repeat mutations among azole-resistant *Aspergillus fumigatus* isolates.

| Strain | ST | Genotype | Location | Collection | Date | *cyp51A* promoter TR status |
| --- | --- | --- | --- | --- | --- | --- |
| F214 | 102 | 10-2-3-5-1-4-2-4-4-2 | Korea | Clinical | 2020-07-24 | TR34 |
| F623 | 106 | 10-2-3-5-2-5-3-11-7-3 | Korea | Clinical | 2022-03-08 | TR34 |
| F669 | 109 | 10-2-3-7-3-4-2-8-3-3 | Korea | Clinical | 2022-06-28 | TR46 |
| F430 | 117 | 10-4-3-5-2-5-4-11-3-3 | Korea | Clinical | 2021-07-15 | TR34 |
| F461 | 123 | 10-6-3-7-3-4-2-7-3-3 | Korea | Clinical | 2021-08-16 | TR46 |
| F296 | 128 | 11-2-5-5-1-4-3-12-4-3 | Korea | Clinical | 2020-12-18 | TR34 |
| NRZ-2017-381 | 132 | 10-2-3-6-1-3-2-9-2-1 | Germany | Clinical | 2017-01-01 | TR34 |
| NRZ-2017-214 | 133 | 8-2-3-3-3-3-2-10-3-2 | Germany | Clinical | 2017-01-01 | TR46 |
| NRZ-2018-529 | 152 | 7-5-3-5-3-5-2-9-4-2 | Germany | Clinical | 2018-01-01 | TR34 |
| NRZ-2017-362 | 155 | 8-6-3-3-3-3-2-6-3-2 | Germany | Clinical | 2017-01-01 | TR34 |
| NRZ-2018-539 | 266 | 7-5-3-5-3-5-2-9-4-2 | Germany | Clinical | 2018-01-01 | TR46 |
| NRZ-2018-290 | 280 | 8-3-3-6-1-3-2-9-3-2 | Germany | Clinical | 2018-01-01 | TR34 |
| CNM-CM8714 | 290 | 8-2-3-5-1-4-2-6-4-2 | Spain | Clinical |  | TR34 |
| C-1-67s-1 | 303 | 12-3-3-5-3-0-2-9-3-1 | Germany | Environmental | 2016-07-05 | TR34 |
| I-1-12-1 | 303 | 12-2-3-5-3-3-3-5-3-1 | Germany | Environmental | 2016-05-20 | TR34 |
| NRZ-2016-121 | 303 | 12-4-4-6-1-0-2-11-3-1 | Germany | Clinical | 2016-01-01 | TR34 |
| C-1-6s-2 | 319 | 9-6-3-5-3-3-3-2-3-1 | Germany | Environmental | 2016-04-08 | TR34 |
| A-3-46s-1 | 322 | 8-5-3-5-3-3-5-6-4-2 | Germany | Environmental | 2016-07-13 | TR34 |
| NRZ-2014-065 | 337 | 8-9-3-3-3-3-3-9-3-2 | Germany | Clinical | 2014-01-01 | TR46 |
| CNM-CM8057 | 339 | 8-2-3-6-1-4-2-6-3-2 | Spain | Clinical |  | TR46 |
| E-1-48-2 | 341 | 8-4-3-6-1-5-3-6-1-2 | Germany | Environmental | 2016-04-12 | TR34 |

**Abbreviations:** MLVA, multilocus variable-number tandem-repeat analysis; ST, sequence type; TR, tandem-repeat.


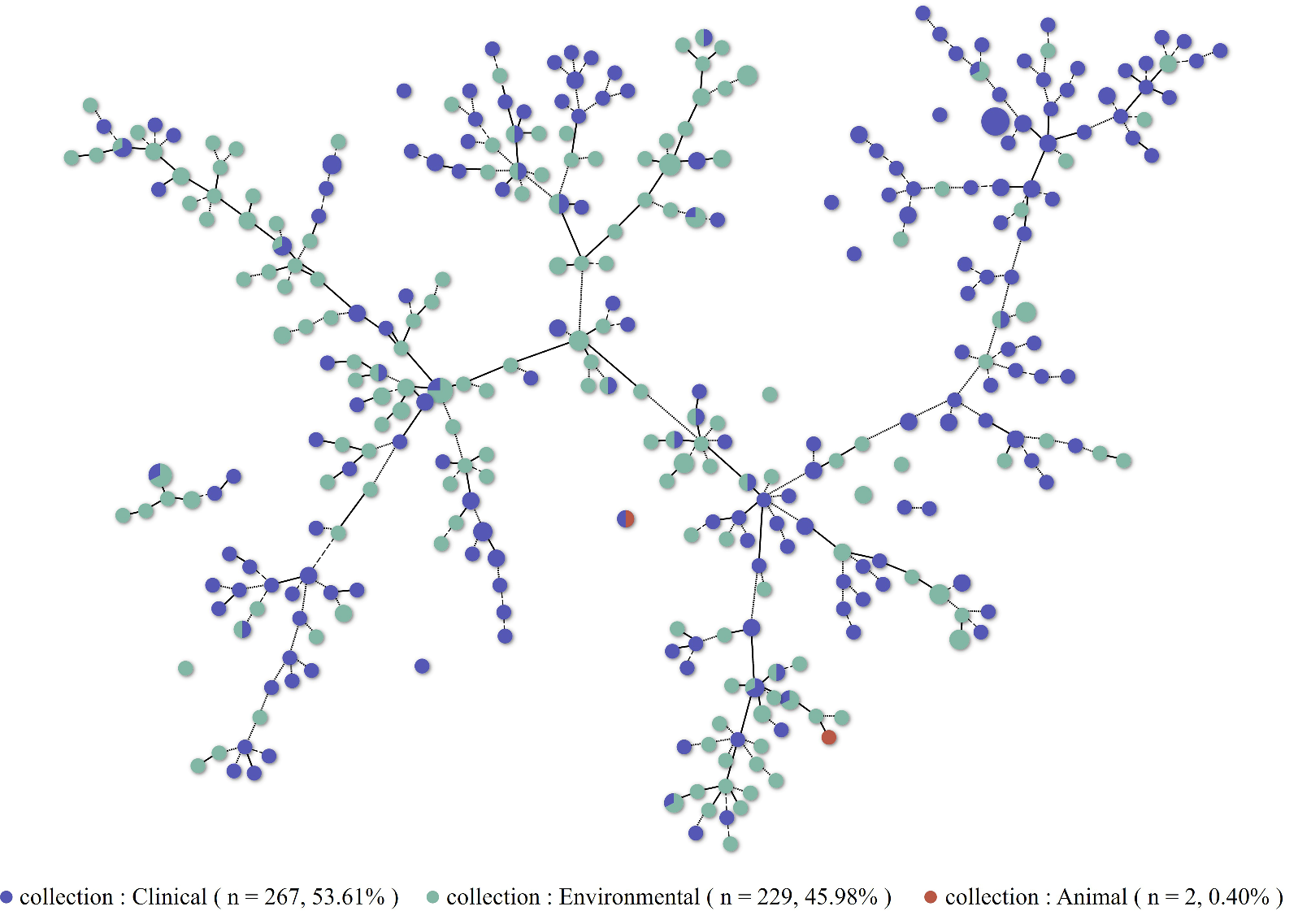
**Figure S1. Minimum spanning tree (MST) of 498 *Aspergillus fumigatus* isolates, color-coded by source of isolation.**

The MST was generated based on multilocus variable-number tandem-repeat profiles. Each node represents a unique sequence type, with node size proportional to the number of isolates. Nodes are colored by source: clinical (blue), environmental (green), and animal (red). Isolates from different sources were broadly intermixed across all clusters, with no evidence of source-specific segregation.


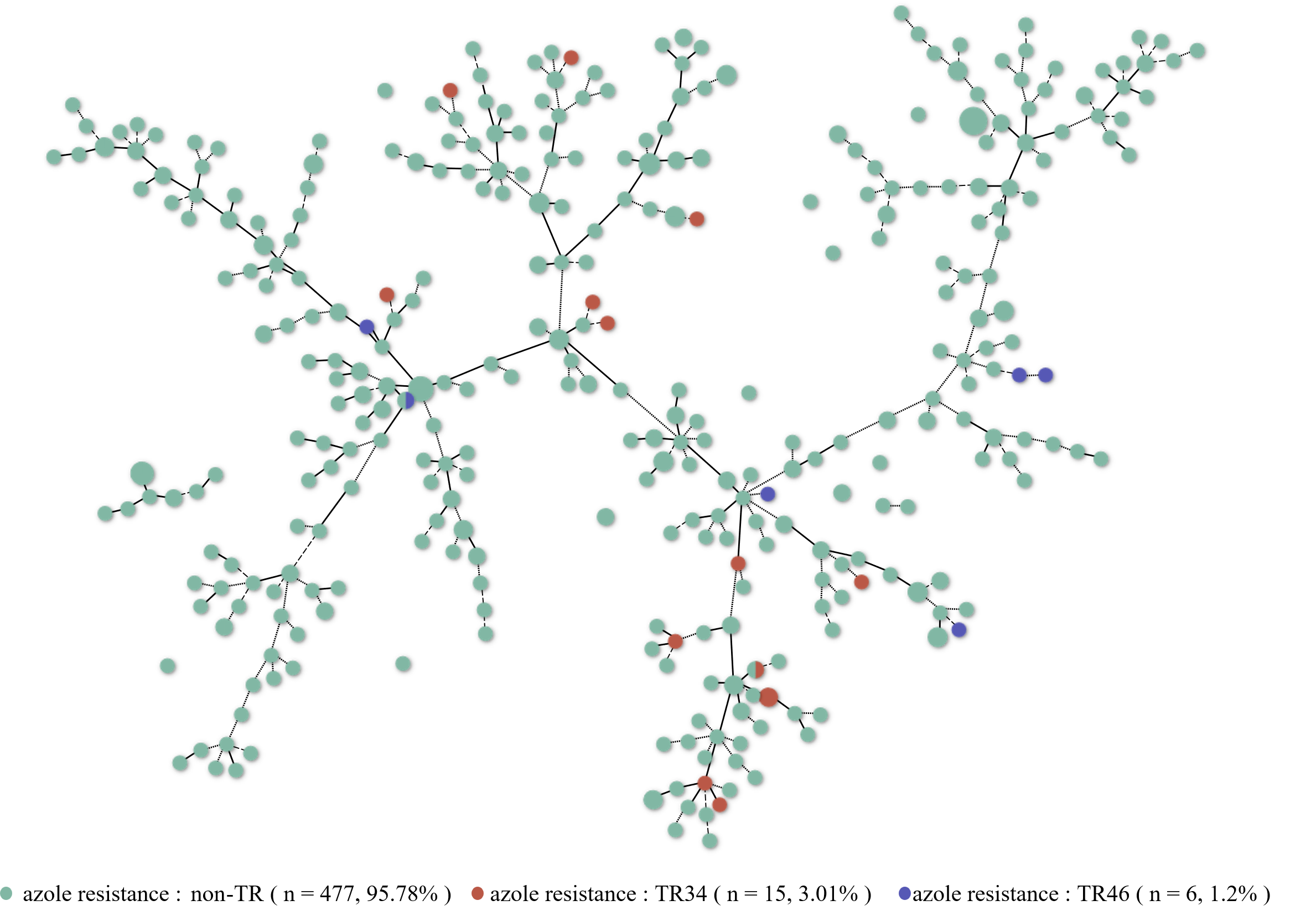
**Figure S2. Minimum spanning tree (MST) of 498 *Aspergillus fumigatus* isolates highlighting *cyp51A* promotor tandem-repeat (TR) mutations associated with azole resistance.**

The MST was constructed from multilocus variable-number tandem-repeat profiles. Each node represents a unique sequence type (ST), with size proportional to the number of isolates. Nodes are colored by *cyp51A* genotype: non-TR (green), TR34 (red), and TR46 (blue). Among 19 resistance-associated STs, 13 (68.4%) were localized within Cluster 3 (predominantly European), while the remaining STs were observed in Cluster 4 and were limited to South Korea, indicating regional aggregation of resistance genotypes..

**Reference**

[1] Lee R, Kim WB, Cho SY, Nho D, Park C, Chun HS, Myong JP, Lee DG (2025) Genetic relationships of Aspergillus fumigatus in hospital settings during COVID-19. Microbiol Spectr 13 (5):e0190224

[2] Lee R, Kim WB, Cho SY, Nho D, Park C, Yoo IY, Park YJ, Lee DG (2023) Clinical Implementation of β-Tubulin Gene-Based Aspergillus Polymerase Chain Reaction for Enhanced Aspergillus Diagnosis in Patients with Hematologic Diseases: A Prospective Observational Study. J Fungi (Basel) 9 (12)

[3] Ambroise J, Bearzatto B, Durant JF, Irenge LM, Gala JL (2025) On the ability to extract MLVA profiles of Vibrio cholerae isolates from WGS data generated with Oxford Nanopore Technologies. BMC Res Notes 18 (1):18

[4] Ambroise J, Irenge LM, Durant JF, Bearzatto B, Bwire G, Stine OC, Gala JL (2019) Backward compatibility of whole genome sequencing data with MLVA typing using a new MLVAtype shiny application for Vibrio cholerae. PLoS One 14 (12):e0225848

[5] Hunter PR, Gaston MA (1988) Numerical index of the discriminatory ability of typing systems: an application of Simpson's index of diversity. J Clin Microbiol 26 (11):2465-2466
